# The Relation between Geometry and Time in Mental Actions

**DOI:** 10.1371/journal.pone.0051191

**Published:** 2012-11-30

**Authors:** Charalambos Papaxanthis, Christos Paizis, Olivier White, Thierry Pozzo, Natale Stucchi

**Affiliations:** 1 Université de Bourgogne, Unité de Formation et de Recherche en Sciences et Techniques des Activités Physiques et Sportives, F-21078 Dijon, France; 2 Institut National de la Santé et de la Recherche Médicale (INSERM), Unité 1093, Cognition, Action et Plasticité Sensorimotrice, BP 27877, F-21078 Dijon, France; 3 Université de Bourgogne, Centre d'Expertise et de la Performance, Unité de Formation et de Recherche en Sciences et Techniques des Activités Physiques et Sportives, F-21078 Dijon, France; 4 Department of Robotics, Brain and Cognitive Sciences, Istituto Italiano di Tecnologia, Genova, Italy; 5 Department of Psychology, University of Milano-Bicocca, Milano, Italy; 6 Institut Universitaire de France, Paris, France; McMaster University, Canada

## Abstract

Mental imagery is a cognitive tool that helps humans take decisions by simulating past and future events. The hypothesis has been advanced that there is a functional equivalence between actual and mental movements. Yet, we do not know whether there are any limitations to its validity even in terms of some fundamental features of actual movements, such as the relationship between space and time. Although it is impossible to directly measure the spatiotemporal features of mental actions, an indirect investigation can be conducted by taking advantage of the constraints existing in planar drawing movements and described by the two-thirds power law (2/3PL). This kinematic law describes one of the most impressive regularities observed in biological movements: movement speed decreases when curvature increases. Here, we compared the duration of identical actual and mental arm movements by changing the constraints imposed by the 2/3PL. In the first two experiments, the length of the trajectory remained constant, while its curvature (Experiment 1) or its number of inflexions (Experiment 2) was manipulated. The results showed that curvature, but not the number of inflexions, proportionally and similarly affected actual and mental movement duration, as expected from the 2/3PL. Two other control experiments confirmed that the results of Experiment 1 were not attributable to eye movements (Experiment 3) or to the perceived length of the displayed trajectory (Experiment 4). Altogether, our findings suggest that mental movement simulation is tuned to the kinematic laws characterizing actions and that kinematics of actual and mental movements is completely specified by the representation of their geometry.

## Introduction

Mental imagery is a cognitive tool that helps humans take decisions by simulating past and future events. Part of this mental process is motor imagery, in which body movements are internally simulated without actually being executed. Neuroimaging and psychophysical investigations have provided robust evidence that mental and actual movements trigger similar motor representations and share overlapping neural substrates [1]–[4]. Notably, common activations of the parietal and prefrontal cortices, the supplementary motor area, the premotor and primary motor cortices, the basal ganglia and the cerebellum were highlighted. Furthermore, autonomic activation increases proportionally to the mental effort produced during imagined movements [5], [6]. Lastly, mental and actual movements preserve the same temporal characteristics [7], [8], while appropriate mental training can enhance motor performance [9]–[11].

Neuroimaging and behavioural studies have contributed to our knowledge about mental events by revealing their neural substrate and by describing their similarities with actual actions, respectively. Despite this, how actions are mentally encoded and replicated remains enigmatic. The most elementary, although universal question, concerns the relationship between space and time. For example, consider a painter who is preparing to draw a figure. Is she able to mentally vary the velocity of her pencil as she does during actual drawing? Or rather, does her action representation encode global aspects of the intended movement, such as the total duration or the shape of the figure? With current neuroimaging techniques and behavioural experiments, we cannot directly probe the kinematics of mental representations. The possibility of measuring only the length of time needed to perform mental movements is an objective limitation to directly investigate their kinematics. However, by recording the total duration of a mental action, one can make robust inferences about its internal structure. For instance, by asking subjects to imagine tracing geometrical figures with different curvatures, while recording their mental time, we can estimate whether actions are internally simulated with constant or variable velocity. In this study, which comprised two main and two control experiments, our aim was to understand the relationship between geometry and time when we don't interact with the physical world, i.e., when we mentally simulate an action. To design our stimuli and formulate our predictions, we took advantage of a robust motor law known as the two-thirds power law (2/3PL) [12], [13]. This law states that the velocity of a planar arm movement is modulated by the local geometry of its trajectory; in other words, the larger the curvature the slower the velocity [14]–[19]. This co-variation between geometry and kinematics is usually expressed as an inverse relationship between the angular velocity *v* and the curvature *k* (*v = αk* ^−1/3^). Our prediction was that in mental movements, as in actual movements, the geometry of the trajectory and movement duration would be tightly coupled.

The key idea of Experiment 1 was straightforward: the 2/3PL states that one should spend more time tracing a tangled trajectory than a straight line. Therefore, for a constant length, movement duration should only depend on curvature. We created four trajectories with same length, but an increasing absolute curvature at their bends. We expected that arm movements actually and mentally tracing these paths would take longer for more curved trajectories. However, if a different rule holds for mental movements, for example constant velocity, we could reasonably assume that only the length would affect mental duration. What we designate as the rule of ‘*constant velocity*’ is nowhere explicitly stated in the literature. Indeed, previous investigations have shown that a linear relationship holds between response time and angle of mental rotation [20], [21] and mental scanning of distances [22]. Note, however, that the amount of path curvature is not the only parameter that could influence movement duration. Changes in mental and/or actual movement durations could be attributed either to a variable attention load in discrete movements [23] or to a greater elaboration requirement when movement direction changes in continuous movements [24]. Experiment 2 was designed to test whether curvature *per se* or attention requirements in changing movement direction modulate the speed of mental movements. Experimentally, the contribution of the resource overload from the co-variation between geometry and kinematics can be disentangled by acting on other time consuming processes taking place during the movement. Besides the local curvature, the inflexions also require attention and elaboration resources. Actually, frequently swinging the direction of a movement from positive to negative curvature, and vice versa, is a reasonably time-consuming process. We therefore designed five trajectories of identical length, identical total cumulative direction change and identical local curvature at curves, but in which the number of inflexions varied between 1 and 5. For these stimuli, the 2/3PL predicts constant movement duration whatever the number of inflexions, because the relevant parameter is the co-variation between curvature and velocity. Therefore, actual and mental durations of the five different trajectories should be independent of the number of inflexions. Finding the opposite would mean that some online time consuming control related to inflexions is taking place during movement representation.

Our findings clearly revealed that duration of mental movements varies with path curvature, which indirectly supports that the 2/3PL holds for mental movements. Note that in two control experiments, we verified that neither oculomotor activity during mental movements (Experiment 3) nor perceptual biases regarding the length of the stimuli (Experiment 4) could influence the interpretation of our results.

## Methods

### Ethical statement

All participants gave their written informed consent prior to their inclusion in this study, which was carried out in accordance with legal requirements and international norms (Declaration of Helsinki, 1964), and approved by the Dijon Regional Ethics Committee.

### Main Experiments

#### Participants

Twelve students (7 men and 5 women; mean age: 22.1±1.9 years) from the University of Dijon participated in Experiments 1 and 2 after giving their written consent. All were in good health, with normal or corrected to normal vision and did not present any neurological, muscular or cognitive disorder. Nine participants were right-handed and three were left-handed according to the Edinburgh Handedness inventory [25]. A French version of the Revised Movement Imagery Questionnaire MIQ-R assessed participants' motor imagery ability [26]. Every participant had a good motor imagery score (mean of 42.3±1.9, out of 56).

#### Apparatus and stimuli

**Figure 1:**
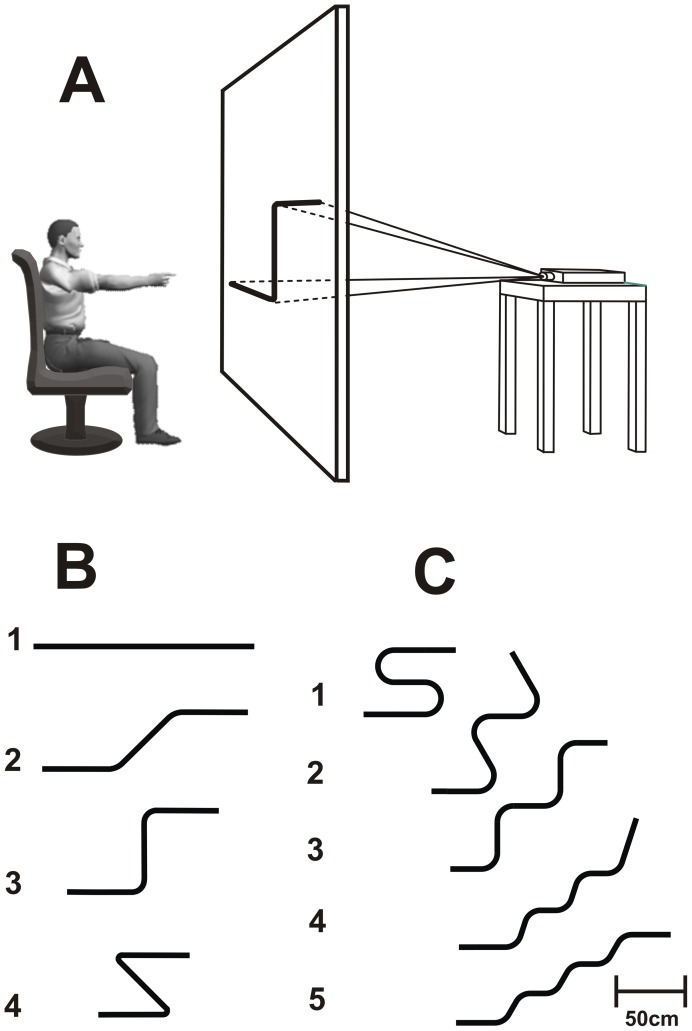
Experimental apparatus and stimuli used in the experiments. (A) Side view of participants' initial position before the execution of an actual movement. (B) Trajectories used for experiments 1, 3, and 4. Trajectories 2, 3 and 4 are characterized by two curves with an increasing curvature. The radius of curvature (defined as the inverse of the curvature) at these curves is 15.2 cm, 8.8 cm, and 2.7 cm, respectively. Trajectories 2, 3 and 4 induced 45deg, 90deg and 135deg changes of directions at each curve, respectively. Thus, the absolute cumulative amount of direction change ranged from 0deg (trajectory 1) to 270deg (trajectory 4). (C) Trajectories used for Experiment 2 and 4. Trajectories in Panels B and C have the same length (160 cm). Additionally, trajectories in Panel C have the same curvature at curves (radius of curvature of 10 cm) and an increasing number of inflexions from 1 to 5. The absolute cumulated changes of direction always summed to 360deg.

The experiments took place in a quiet room (4×6 m), temperature regulated at 22±2°C and illuminated with homogeneous white light. A rear projection system was used for the experiments. A vertical panel (height: 2 m; width: 1.5 m) shaped with white textile was placed in the middle of the room (see Fig. 1A). A video projector (Epson emp1815 LCD, 70 Hz, 1280×800pixels) placed 2 m behind the screen was used to retro-display the stimuli. Participants were comfortably seated on a chair 1.4 m in front of the screen. The position of the projected stimuli on the panel was adjusted to each participant's height so that her eyes were at the same level as the stimulus. Templates of movement trajectories comprised either four lines of equivalent length (160 cm, 1 cm thick), but increasing curvatures at bends in the trajectory: 0, 1/15.2, 1/8.8, and 1/2.7 cm^−1^ (Experiment 1, see Fig. 1B), or five lines of equivalent length (160 cm) and curvature at bends 1/10 cm^−1^, but an increasing number of inflexions (Experiment 2, see Fig. 1C). Movement direction along these trajectories, defined as the geometrical tangent to the current position, changes with curvature.

#### Procedure

In Experiment 1 we investigated whether mental movements obey the 2/3 PL. The experiment consisted of a *mental-movement* session followed by an *actual-movement* session, separated by two days. To avoid any influence of actual movements on mental movements, all participants started with the mental session. For the sake of clarity let us introduce the actual session first. Once the trajectory had been displayed, participants pointed their index fingertip to either end of the line. Starting positions were randomly chosen between the left (50%) and right ends of the trajectory. The arm was fully extended, the semi-pronated hand was in axis with the upper arm and the forearm, and the four other fingers were closed. After a ‘go’ signal provided by the experimenter, the participant traced the trajectory with her index finger without touching the screen. No specific instructions were provided regarding eye movements, but participants were instructed to avoid head movements. Relatively long trial durations are necessary to obtain reliable measurements in motor imagery protocols because movement durations have a coarse resolution [2], [9], [11], [27]. Therefore, in our protocol one trial corresponded to three successive and fluid tracing movements at a spontaneous pace (for instance, when starting from the left end, the participants performed rightward-leftward-rightward movements). Each participant carried out 10 trials for each trajectory.

The same experimental procedure was adopted for mental movements. When a trajectory appeared on the screen, participants were asked to imagine their arm in the same configuration as in actual movements pointing to the left or right end of the trajectory. We emphasized to participants that they had to feel themselves performing the task (motor or first-person perspective). Imagining a movement in the first person is a necessary condition to engage the motor system [28]. Note that during mental movements, participants' arms were motionless upon their knees. Once the participant had a clear mental image of the required initial arm position, she said ‘prêt’. A ‘go’ signal was given after 2–3 seconds and the participant mentally traced the displayed trajectory at a natural speed. As for actual movements, one trial corresponded to three successive fluid mental tracing movements, and 10 replications for each trajectory were carried-out. In both actual and mental sessions, participants rested one minute to avoid physical fatigue after 8 consecutive trials. The presentations of trajectories were counterbalanced across participants. Each session lasted about 30 minutes. We recorded and analyzed the activity of the deltoid muscle during mental trials (see below). Before each experimental session, participants performed 12 practice trials. A circle and a parallelogram, which were not used as stimuli in the experiment, were chosen as training trajectories. During the actual practice trials, a laser-pointer attached to participants' index finger provided a visual feedback of the executed movements.

Experiment 2 was carried out one month after Experiment 1 and involved the same twelve participants and procedures. Here, we tested the possibility that attention, and not geometry, may affect the duration of mentally simulated arm movements. We used five trajectories, which differed from each other only in the number of inflexions (i.e., points on a curve at which the curvature changes concavity) increasing from 1 to 5 (see Fig. 1C). The other salient geometrical aspects, which are the trajectory length (160 cm), the curvature at bends in the movement path (curvature equal to 1/10 cm^−1^), and the cumulative absolute angular direction change (equal to 360 deg), were kept strictly constant. As in Experiment 1, the mental session always took place before the actual session. We also recorded and analysed the activity of the deltoid muscle during mental trials (see below).

### Control Experiments

The first control study (Experiment 3) was carried out one month after Experiment 2 and replicated the first experiment with the difference that participants fixated the centre of the stimulus during mental and actual movements. Therefore, any change in the duration of mental arm movements with path curvature should be attributed to path curvature and not to any eye movement strategy. Eight students (5 men and 3 women; mean age: 24.4±2.3 years) from the University of Dijon participated in Experiment 3 after giving their written consent. Half of them had already participated in Experiment 1. All were right-handed, in good health, with normal or corrected to normal vision, and did not present any neurological, muscular, or cognitive disorder. EOG signals were recorded to verify online that eyes remained motionless (as in [29]). Four silver-chloride electrodes were positioned on the lightly abraded skin near the lateral corner of each eye. EOG signal gain was set at 500, and low pass filtered at 10 Hz. Data were sampled at 500 Hz and digitalized using the Biopac MP150 (Biopac system, CA, USA). Trials showing EOG activity were performed again (14 actual trials out of 400 (3.5%) and 12 mental trials out of 400 (3%)).

In the second control study (Experiment 4), carried out two months after Experiment 3, we addressed the possibility that perceived trajectory length may influence movement duration, in addition to curvature. Twenty students from the University of Milano-Bicocca (7 men and 13 women; mean age: 22.4±1.4 years), all with normal or corrected to normal vision, participated in this experiment. None of them had participated in the previous experiments. The nine visual stimuli in this experiment were the trajectories used in Experiments 1 and 2. All these trajectories have the same length, but they appear to have different lengths. Participants were asked to scale the stimuli by using Thurstone's method of pair comparisons. The 36 possible couplings of the stimuli were presented in a random order on a computer screen. Participants were asked to decide which of the two stimuli was the longest. The whole scaling procedure lasted less than 10 minutes for each participant.

#### Movement timing recording and statistical analysis for Experiments 1, 2 and 3

Movement duration is a key variable that can be measured during mental movements and compared with actual movements. In this study, we applied the method of mental chronometry, which provided reliable and consistent results in several studies [11], [30]. By means of an electronic stopwatch (temporal resolution 1 ms), manipulated by the experimenter, we measured actual and mental movement durations, which were defined as the elapsed time between the experimenter's go signal and the participant's stop signal.

The experimental design of Experiments 1 and 3 consists of three within-subjects factors: *session* (2 levels: mental and actual movements), *trajectory* (4 levels, see Fig. 1B) and *replication* (10 levels), for a total of 80 trials (2×4×10). The design of Experiment 2 was equivalent to that of Experiment 1 except that the *trajectory* factor had 5 levels (see Fig. 1C) therefore leading to 100 trials in total (2×5×10). All variables showed normal distributions (Shapiro-Wilk tests) and, therefore, statistical effects were assessed with repeated measures analyses of variance (ANOVA). Post-hoc analysis was performed with Bonferroni's tests. Significance level was set at alpha = 0.05.

#### EMG recording and analysis for Experiments 1 and 2

During mental sessions, the EMG activities of the three heads of the deltoid muscle (the anterior, AD, flexor of the shoulder joint; the lateral, LD, abductor of the shoulder joint and the posterior deltoid, PD, extensor of the shoulder joint) were recorded (Biopac MP150, Biopac system, CA, USA). Two silver-chloride surface electrodes of 10-mm in diameter were positioned on the muscle belly with an inter-electrode distance (centre to centre) of 2 cm. The reference electrode was placed on the left ankle. EMG signals were recorded at a frequency of 1000 Hz, band pass filtered (20–400 Hz) and stored for off line analysis using MATLAB® routines.

The root mean square 
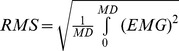
 of EMG signals was used to quantify the activation level of the deltoid muscle (MD represents the movement duration). We decided to analyse the EMG activity of all mental trials within the temporal window between 10% and 90% of the whole movement acquisition (as in [31]). In this way, we were certain that participants had already started, but still not finished their mental trials. In order to quantify muscle activation, we compared RMS during mental trials with RMS recorded from the same muscle totally relaxed during a rest period (10 trials of 7 s per participant).

## Results

### Experiment 1: Does curvature affect the duration of mentally simulated arm movements?

**Figure 2:**
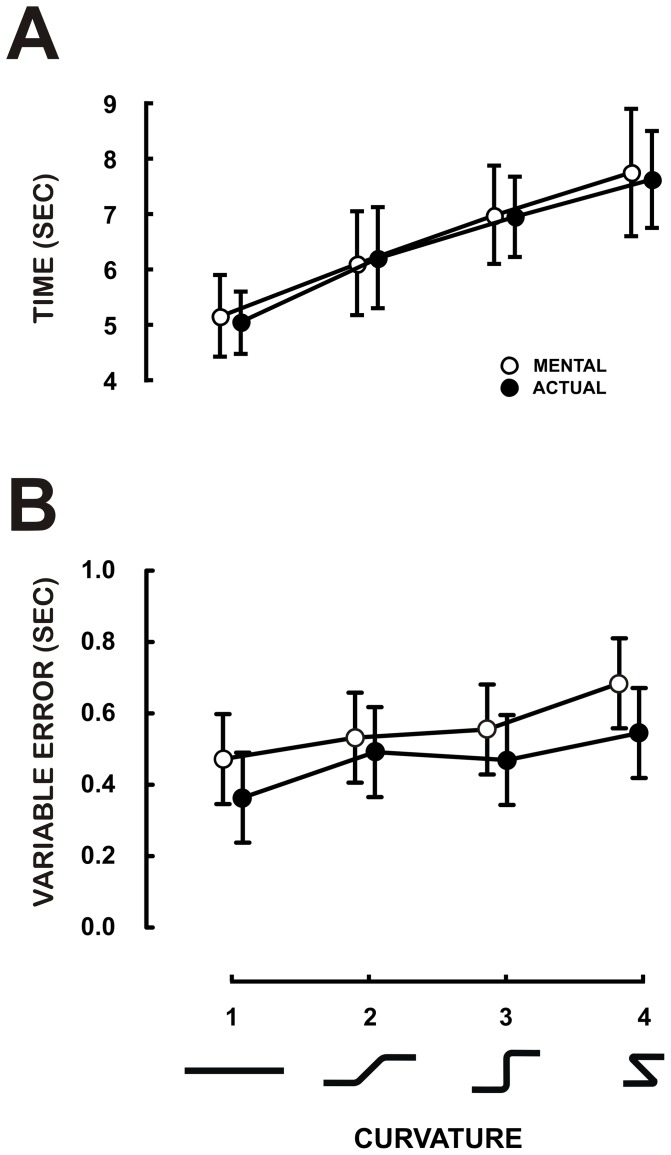
Experiment 1: Relation between curvature and duration. The shapes below the x-axis reproduce the trajectories used as templates for movements (see Fig. 1B). (A) Mean duration of actual and mental movements computed over 12 participants. (B) Variability over replications. The disks correspond to the mean SDs over subjects. The error bars represent the 95% Confidence Interval.

**Figure 3:**
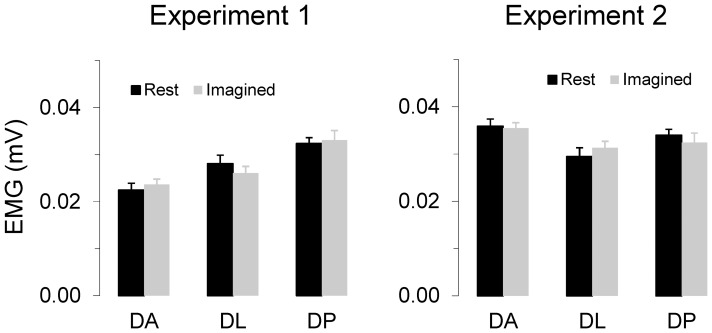
Experiment 1 and 2: Muscle activation during mental movements. Average (± SD) RMS values of the EMG activity of the three heads of the deltoid muscle for both mental and rest conditions in the two experiments. AD: anterior deltoid, LD: lateral deltoid, PD: posterior deltoid.

Figure 2A shows average durations with their 95% confidence intervals for mental (open circles) and actual (closed circles) arm movements for the four trajectories. All movement durations significantly increased with trajectory curvature (F_3,33_ = 46.56, MS = 292.25, p<0.001). The average durations (±SD) from the smallest to the greatest curvatures were 5.1±0.98 s, 6.25±1.3 s, 6.97±1.22 s, and 7.69±1.55s, respectively. Interestingly, durations of actual and mental arm movements closely matched together. This fact is further confirmed by the absence of statistical significance in the factor *session* (F_1,11_ = 0.06, MS = 0.27, p = 0.81). The ANOVA did not report any other significant effect, including interaction (p>0.05). Each movement was repeated 10 times. We quantified the participants' precision to reproduce actual and mental movements by calculating the individual standard deviations (SD) computed over replications. Figure 2B shows the mean SD (Variable Error) plotted against the four trajectories, computed over subjects for the mental (open circles) and actual (closed circles) movements. ANOVA revealed a main effect of trajectory (F_3,33_ = 5.29, MS = 0.15, p<0.01). Although this result is difficult to interpret given the tiny variability interval of the means (which are comprised between 0.4 and 0.6 s), it clearly appears that the precision is rather good relative to the mean duration of movements (see Fig. 2A). In other words, participants were able to reproduce the same movements in both modalities with a mean variability over replication of less than 10% of the mean duration. Fig. 3 (Experiment 1) shows the average RMS values from the deltoid muscle (anterior deltoid, lateral deltoid and posterior deltoid) during rest and mental movements. ANOVA on RMS values with two within-subject factors (the three heads of the deltoid muscle crossed with the two conditions of mental and rest trials) revealed that the deltoid activation patterns did not show any significant difference between rest and mental movements (F_1,11_ = 0.21, MS = 28×10^−6^, p = 0.64), which is consistent with the previous findings of our group [6], [27], [31].

Experiment 1 indirectly shows that we are able to modulate the duration of a mental movement according to its geometry as for actual movements. In previous experiments, changes in movement duration were attributed either to a variable attention load in discrete movements [23] or to a greater elaboration requirement when the movement direction is changing in continuous movements [24]. In other words, these investigations might suggest that a change of direction may induce an increase in attention resources and, thus, longer durations. The next experiment was designed to address this question.

### Experiment 2: Does attention affect the duration of mentally simulated arm movements?

**Figure 4:**
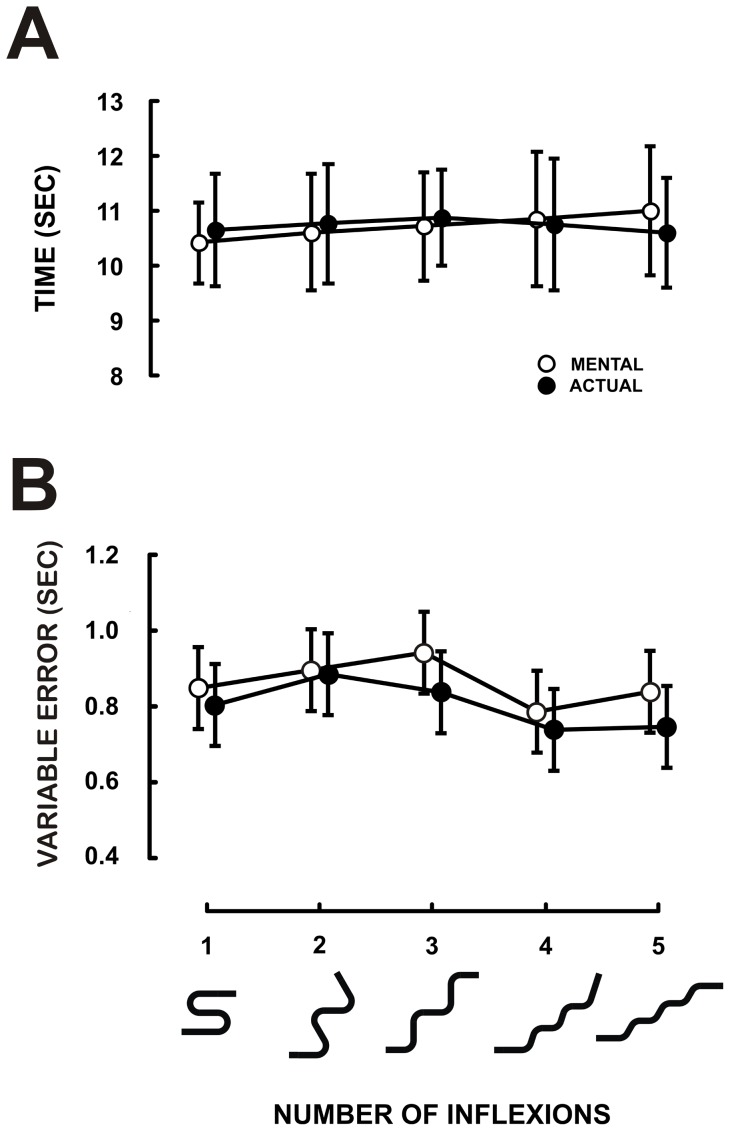
Experiment 2: Relation between number of inflexions and duration. The shapes below the x-axis reproduce the trajectories used as templates for movements (see Fig. 1C). (A) Mean duration of actual and mental movements computed over 12 participants. (B) Variability over replications. The disks correspond to the mean SDs over subjects. The error bars represent the 95% Confidence Interval.

Figure 4A shows the average duration with its 95% confidence interval for mental (open circles) and actual arm movements (closed circles) as a function of the number of inflexions of the trajectories. ANOVA failed to report any significant effect of session (actual vs. mental: F_1,11_ = 0.005, MS = 0.001, p = 0.94) or trajectory (F_4,44_ = 0.16, MS = 3.1, p = 0.96) on movement duration. This clearly confirms that the number of inflexions does not influence actual or mental movement durations. Figure 4B depicts the mean SD of duration (Variable Error). As in the previous experiment, the overall precision is quite good. ANOVA didn't report any main or interaction effect on variable error. Muscle activation during motor imagery did not differ from the baseline condition (F_1,11_ = 37×10^−2^, MS = 12×10^−6^, p = .55; see Fig. 3, Experiment 2).

The outcomes of Experiment 2 strongly support the first interpretation of the results; that is, as for actual movements, the increasing durations of mental movements are mainly caused by the increasing trajectory curvature, and not its number of inflexions. However, before drawing definitive conclusions, we need to verify that our results are independent of oculomotor activity. Previous studies have found that smooth pursuit eye movements were modulated by curvature. In other words, they obeyed the 2/3PL [32]. Therefore, eye movements can potentially guide the modulation of mental movement durations with path curvature. In control Experiment 3, we asked another set of participants to replicate Experiment 1 and we instructed them to keep their eyes motionless.

### Experiment 3: Do eye movements affect the duration of mentally simulated arm movements?

**Figure 5:**
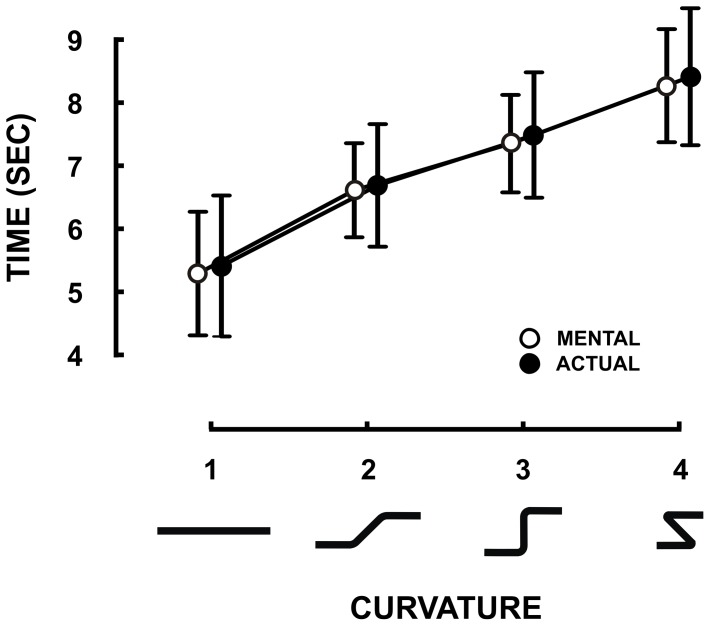
Experiment 3: Relation between curvature and duration in fixation condition. (A) Mean and 95% Confidence Interval of durations of actual and mental movements (N = 8).

Figure 5 shows average values for mental and actual arm movement durations as a function of the curvature of the trajectories (defined in Fig. 1B). As in Experiment 1, ANOVA only reported a main effect of trajectory (F_3,21_ = 65.14, MS = 253.87, p<0.001). *Post hoc* comparisons revealed that all movement durations increased with curvature (p<0.05 for all comparisons). This control experiment strengthens results from Experiment 1 and rules out any clear influence of oculomotor strategy on the covariation between duration and curvature of trajectory.

Before showing the results of our last experiment, let us consider its rationale. Perhaps the reader will have already perceived from Fig. 1 that the lengths of the trajectories used in the previous experiments appear to be different, although they are strictly the same. This visual illusion, which is worthy of study in and of itself, probably depends on the number of curves and their curvature: the more tangled a string the shorter its perceived length. Although the mechanisms behind this illusion are beyond the scope of this investigation, its existence could influence the interpretation of our results. One of the main points of the argument supporting our study rests on the geometrical constant length of the trajectories we used as visual templates for the imagined movements. The fact that these trajectories do not appear to be the same length undermines our argument to its foundations. If we find a correlation between the perceived length of the four trajectories used in Experiment 1 and the duration of the corresponding imagined movements, the duration trend we found in Experiment 1 would lose any interest for the central thesis of our paper. The analogy between the durations of actual and imagined movements shown in Experiment 1 is a very strong datum but logically insufficient to attribute a modulation of velocity to the imagined movement if the lengths of the visual trajectories used as templates are perceived as different. Consequently, testing whether movement durations increase with the perceived length of the stimulus, and not only the curvature of the trajectory, is addressed in the last control experiment.

### Experiment 4: Does perceived trajectory length influence movement duration?

**Figure 6:**
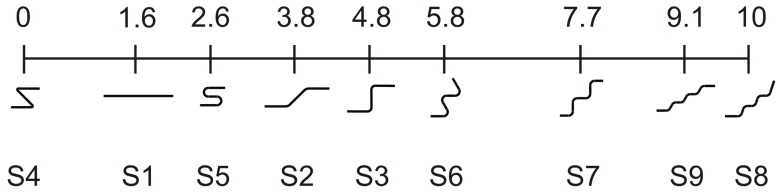
Experiment 4: Scale of the perceived length. Perceptual scaling of the 9 visual stimuli obtained with the Thurstone procedure (arbitrary units).

Scale values were attributed to the apparent length of each trajectory by using Thurstone's method of pair comparisons [32], [33] (see Methods). Then, the correlation between the scale values of the trajectories and their corresponding durations was computed. A null correlation was expected for the trajectories of Experiment 1 (Fig. 1B), which have yielded a clear trend of the durations. The Thurstone procedure allows for computing the scale values of the 9 stimuli on a linear scale which has the properties of an interval scale. In particular, we used the so called Thursone's case V adapted for the presence of extreme proportions. In the end, we obtained the psychological scale reported in Figure 6. The units of the scale are arbitrary, but the distances between the values are meaningful. This scale shows that a perceived illusory effect exists even if it does not allow us to establish its size, which can range from large to very small. Even a visual inspection of the scale values of stimuli S1-S4 compared to the corresponding durations obtained in the first experiment clearly shows that the values are uncorrelated (r = −0.14, p = 0.85). Rather, the correlation is high (r = .96, p<0.001) between S5–S9 and their corresponding durations (Experiment 2). However, this result is meaningless given that the durations were constant. Therefore, any illusory effect that explains the modulation of duration with trajectories used in these experiments can also be ruled out.

## Discussion

Together, the results of the main experiments show that durations of mental movements must be attributed to the shape of their trajectory, just like for actual movements (Experiment 1). This statement is a direct consequence of how our stimuli were conceived. In our conditions, duration reflects the mean velocity of the movements because their trajectory length is constant. In our actual movements, the difference in mean velocities between the various stimuli is the consequence of the modulation of velocity caused by the changing curvature, as could be predicted by the 2/3PL. Given the close similarity between mental and actual durations, our results show that mental movements are also generated with a changing velocity tuned to the movement shape. Our data do not allow us to claim that the covariation between curvature and velocity in mental movements follows the 2/3PL, but the suggestion in this direction is quite strong. In compliance with the 2/3PL, the number of inflexions has no effect on duration for either mental or actual movements (Experiment 2). The two control studies (Experiments 3 and 4), which tested for possible alternative explanations, strengthen the results of the main experiments. Experiment 3 showed that the relation between geometry and time was independent of eye movements. Experiment 4 demonstrated that the time needed for tracing the trajectories was not correlated with their perceived lengths.

### Kinematic laws for mental and actual actions

We found a close correspondence between mental and actual movement durations. This is in agreement with previous studies showing that mental action simulation and its actual execution share common neural and cognitive processes [4], [8], [11], [34]. This main finding broadens and completes the conclusions of previous investigations which demonstrated that Fitts' law holds for mental actions [7], [35], [36]. Fitts' law predicts the total movement duration according to target size and movement distance; i.e., precision constraints and movement time increase concomitantly. Here, we show, for the first time, that geometry determines the duration of mental actions. This finding indicates that actions are internally simulated with varying velocity, and suggests that the 2/3PL may hold for imagined movements. Overall, the fact that imagined movement obeys precision (Fitts' law) and geometrical constraints indicates that mental events are precisely tuned to laws of kinematics like actual movements.

Our findings were not easily anticipated. One could reasonably assume that actions are mentally represented in a somewhat simplified form. A careful inspection of the literature regarding perception/mental-imagery coupling provides arguments for such a hypothesis. The very first studies on the mental rotation of objects [20], [21] and on scanning of mental images [22] seem to confirm this assumption by suggesting that motion is imagined at constant velocity. Moreover, it has been shown by matching eye movements and mental chronometry that we are able to imagine circular motion at constant speed [37]. The possibility that only constant speed is used in mental imagery is a plausible hypothesis. However, our results rule this hypothesis out as we present a clear counterexample of mental motion at variable speed.

The involvement of the same internal predictive models in both actual and mental movements explains why both are governed by similar kinematic laws [9], [11], [27], [38]. At the neural level, the 2/3PL during actual movements is reflected in the activation of motor and parietal areas [39]. As both mental and actual movements trigger similar brain areas (Jeannerod 2001), one could propose that activation of motor and parietal cortices during mental imagery also reflects a tight correspondence between geometry and time, as in the 2/3 PL.

The 2/3PL we used to design the geometrical shape of our stimuli is a model of trajectory formation, perhaps one of the fundamental problems in motor control [40]. Even if our results do not specifically show that the 2/3PL holds for mental movements, but only that movements are internally simulated with varying velocity, they have some interesting consequences for its interpretation. Several attempts have been made to explain the origin of this law as a consequence of strategies of motor planning. Among them, we can retain those based either on maximizing the smoothness of the movement [41], [42] or minimizing the variance of the effector's final position [43]. Yet, at a more abstract level, we can ask what the nature of movement representation that is implied by the 2/3PL is. One of the most appealing suggestions of the 2/3PL is that planning a movement coincides with deciding its geometrical shape, whereas its kinematics is a necessary and automatic consequence of that decision. Claiming that we must care only about the shape of an upcoming movement still does not specify at which point of movement processing we determine how a movement is actually carried out. To simplify matters, we can envisage two alternative situations: the kinematics is settled either before the beginning of the movement or throughout its execution.

Experiment 2 suggests excluding the second alternative since time consuming processes, such as attention and control of movement direction, do not affect movement duration. The main claim of the first alternative is that shape and movement are deeply tied. A specific geometrical shape of the trajectory is conceived to be potentially traced even though it does not actualize in an overt movement. We can hypothesize that the representation of shapes in our mind is not static but dynamic, because we represent movement trajectories as if they ought to be executed. A similar view has been put forward in the numerous studies on the elusive phenomenon of the so called memory representational momentum where memory for position is distorted in the direction of the implied motion [44]–[47]. Moreover, the first alternative fits well with our specific visual and kinaesthetic sensitivity to the 2/3PL [48]–[50]. Finally, this hypothesis is compatible with the intriguing idea that a planar arm movement complying with the 2/3PL is equivalent to moving at a constant equi-affine speed [17]. Hence, since the planar arm movement turns out to be the simplest motion, the equi-affine reference could be interpreted as an optimal representation of the movement. Our main result that the geometrical shape of a mental movement determines its duration is a further element supporting the first alternative.

### The hypothesis of attention load and time consuming process in the relation between movement shape and duration

Processes, which are set up during movement execution, could modulate movement speed by consuming time and resources differently. Such a hypothesis has been advanced by Pellizzer and Zesiger [24] in an effort to explain the 2/3PL by some functional constraints. Echoing the classical results on mental rotation, the authors suggested that the transformation of the intended direction of a mental or actual planar arm movement is performed by a process consuming a length of time that is directly proportional to the angle of rotation [21]. In addition, recent findings [23] have shown that the expected isochrony, isometry, and equal kinematics of mental and actual movements fail for plain discrete arm movements. Therefore, the functional equivalence between mental and actual actions could hold only for movements that are complex enough to require a demanding attentional load.

With Experiment 2, we have excluded the possibility that the relation between duration and geometry is to be attributed to resource consuming processes that take place during both mental and actual movements. In particular, neither the attention request nor the computational load, which are both supposed to intensify at the abrupt changes in movement direction, have an effect on movement duration. Rather only the local curvature of the movement trajectory decides its speed.

### Central or peripheral origins for the relation between movement shape and duration

Experiments 1 and 2 have some broader consequences which deserve one last brief comment. Our primary aim was to show that a relation between movement shape and duration holds for mental movements. Since this relation is preserved in the lack of actual arm movement, thus avoiding the potential artifacts of biomechanical and peripheral factors, it is rational to infer that its origin is rooted in the central stages of motor planning. In other words, a relationship between shape and duration is already present in intended actions, i.e., before choosing the effector or the specific parameters of the movement. One possibility is that the relation between movement shape and duration is a consequence of the covariation between geometry and velocity described by the 2/3PL. Central processes and/or peripheral constraints have been invoked to explain the 2/3PL. Peripheral constraints, such as the forces implied in muscle mechanics [41] and the oscillatory nature of joint rotation [51], [52], have been proposed. However, they are in contrast with several behavioural lines of evidences showing that the 2/3PL keeps applying in absence of movement [53], with effectors like the eye and the jaw, and even the whole body owing mechanical characteristics that are different from the arm [54]–[56]. Besides, there is a specific perceptual sensitivity to the 2/3PL suggesting an intermodal competence that is hardly compatible with its peripheral origin [50]. Finally, single-cell recording in monkeys [14], [15] and fMRI studies in humans [39] provide further evidence for the origin of the 2/3PL in the central planning and control of the movement. The main result of our study goes in the same direction since a relation between geometry and velocity, even if we still do not know if it is the 2/3PL, holds for mental movements, hence without physical movement of the arm.
